# Effect of *Enterococcus faecium* NCIMB 10415 on Gut Barrier Function, Internal Redox State, Proinflammatory Response and Pathogen Inhibition Properties in Porcine Intestinal Epithelial Cells

**DOI:** 10.3390/nu14071486

**Published:** 2022-04-02

**Authors:** Nikolett Palkovicsné Pézsa, Dóra Kovács, Péter Gálfi, Bence Rácz, Orsolya Farkas

**Affiliations:** 1 Department of Pharmacology and Toxicology, University of Veterinary Medicine Budapest, 1078 Budapest, Hungary; kovacs.dora@univet.hu (D.K.); galfi.peter@univet.hu (P.G.); farkas.orsolya@univet.hu (O.F.); 2 Department of Anatomy and Histology, University of Veterinary Medicine Budapest, 1078 Budapest, Hungary; racz.bence@univet.hu

**Keywords:** *Enterococcus faecium* NCIMB 10415, *Escherichia coli*, *Salmonella* Typhimurium, IPEC-J2, paracellular permeability, ROS, proinflammatory cytokines, adhesion

## Abstract

In farm animals, intestinal diseases caused by *Salmonella* spp. and *Escherichia coli* may lead to significant economic loss. In the past few decades, the swine industry has largely relied on the prophylactic use of antibiotics to control gastrointestinal diseases. The development of antibiotic resistance has become an important issue both in animal and human health. The use of antibiotics for prophylactic purposes has been banned, moreover the new EU regulations further restrict the application of antibiotics in veterinary use. The swine industry seeks alternatives that are capable of maintaining the health of the gastrointestinal tract. Probiotics offer a promising alternative; however, their mode of action is not fully understood. In our experiments, porcine intestinal epithelial cells (IPEC-J2 cells) were challenged by *Salmonella* Typhimurium or *Escherichia coli* and we aimed at determining the effect of pre-, co-, and post-treatment with *Enterococcus faecium* NCIMB 10415 on the internal redox state, paracellular permeability, IL-6 and IL-8 secretion of IPEC-J2 cells. Moreover, the adhesion inhibition effect was also investigated. *Enterococcus faecium* was able to reduce oxidative stress and paracellular permeability of IPEC-J2 cells and could inhibit the adhesion of *Salmonella Typhimurium* and *Escherichia coli*. Based on our results, *Enterococcus faecium* is a promising candidate to maintain the health of the gastrointestinal tract.

## 1. Introduction

Intestinal diseases caused by *Escherichia coli* (*E. coli*) and *Salmonella* spp. may lead to significant economic loss in food-producing animals and may also pose a threat to human health as (1) both bacteria are zoonotic, (2) they may contaminate pork products in the food chain, and (3) they may develop resistance to antibiotics, thus contributing to the transmission of antimicrobial resistance [1,2,3].

To control gastrointestinal diseases, the swine industry has largely relied on the prophylactic use of antibiotics. Due to the growing concern about antibiotic resistance, the use of antibiotics as growth promoters were banned in the European Union in 2006. The new EU regulation (2019/6 of the European Parliament and of the Council of 11 December 2018 on veterinary medicinal products and repealing Directive 2001/82/EC) has come into force on 28 January 2022, further restricting the application of antibiotics in veterinary use [4]. However, according to the One Health concept, antimicrobial resistance is not only a concern for the veterinary sector, but it also affects humans and the natural environment that animals and humans share [5]. Any option that can reduce the spread of resistance is crucial for human health so that antibiotic treatment can remain effective. Finding alternatives capable of maintaining the health of the gastrointestinal tract without the use of antibiotics is not only pivotal for the swine industry, but also for human health [1]. Phytochemicals, pre- and probiotics, organic acids, enzymes, antimicrobial peptides, anti-bacterial virulence drugs, minerals and bacteriophages are nowadays being considered alternatives to antibiotics [6].

The gastrointestinal tract is the main source of reactive oxygen species and is constantly exposed to the luminal environment. If the barrier function is disrupted (due to weaning, changes in the diet/energy balance, immune response) the intestine becomes more vulnerable to oxidative stress, and numerous disorders can develop. Pathogens activate through multiple ways the secretion of proinflammatory cytokines, thus conferring also to oxidative stress [7,8,9,10,11].

Probiotics are “*live microorganisms which when administered in adequate amounts confer a health benefit on the host*” and can exert their beneficial effect in multiple ways [12,13]. Furthermore, modulation of the immune system of the host, a direct effect on the microbiome and the release of microbial products are the most common modes of action [14]. Pre-treatment with probiotic bacteria has the potential to reduce cell death and cell dissociation and retain structural integrity when exposed to invading pathogens [15]. Probiotics modulate heat shock proteins and cytokines, both involved in diverse regulatory pathways [3]. Some probiotic bacteria show antioxidant properties; however, the antioxidant mechanism of probiotics seems to be very complex, and it varies among species. Lactobacillus strains, for example, induce antioxidative enzymes [10]. The probiotic strain, *Lactobacillus reuteri* I5007, has been shown to enhance barrier function through the induction of the abundance of TJ proteins in newborn piglets [7]. Studies revealed that probiotics may also have an inhibitory effect on pathogen adhesion. *Lactobacillus plantarum* ZLP001 and *Lactobacillus reuteri* LR1 inhibited enterotoxigenic *E. coli* (ETEC) adhesion to intestinal mucosa [16,17]. However, the exact mechanism of probiotic action is still unknown.

Most probiotic bacteria are of intestinal origin and belong to a group of lactic acid-producing bacteria, e.g., Bifidobacteria, Lactobacilli and Enterococci [3]. On the one hand, Enterococci are widely used as probiotics to enhance the microbial balance of the intestine but on the other hand, Enterococci are nosocomial pathogens causing bacteraemia, endocarditis, urinary tract and other infections and the multi-drug resistance of Enterococci raises serious concerns [18,19]. The beneficial effect of *Enterococcus faecium* (*E. faecium*) NCIMB 10415 on the immune system and on growth promotions was proved by in vitro [20,21] and in vivo [22,23] experiments. *Enterococcus faecium* HDRsEf1 (Ef1) demonstrated the potential to protect enterocytes from an acute inflammatory response and to strengthen the intestinal barrier against ETEC [24]. Supplementing mice with *Enterococcus faecium* KH 24 strain resulted in beneficial effects, such as better weight gain, a decrease in *Salmonella enteritidis* and coliform colonization, and an increase in Lactobacilli growth [25]. *Enterococcus faecium* NCIMB 10415 is licensed as a feed additive and is currently in use in farm animals, including sows and piglets. It is also beneficial in reducing diarrhea by enhancing the barrier function [3,20].

The IPEC-J2 cell line is a well-characterized, non-carcinogenic cell line originating from the jejunum of piglets [26,27]. Due to the similarities between the pig and human intestine, the IPEC-J2 cell line is not only important for mimicking the GIT of swine but conclusions can also be made for humans [27]. It is a widely used tool for studying the effects of probiotic applications [20,24,28] and other substances (for example proantocyanidines and wheat germ extract) [1,29].

The aim of this study was to examine the potential beneficial effects of *E. faecium* NCIMB 10415 upon pathological challenge induced by two representatives of GI infection-causing agents, *E. coli* or *Salmonella enterica* ser. Typhimurium (*S.* Typhimurium). We hypothesize that pre-, co-, and post-treatment with *Enterococcus faecium* NCIMB 10415 may be beneficial to the intracellular redox state, paracellular permeability, IL-6 and IL-8 secretion of IPEC-J2 cells, and affects adhesion properties of *E. coli* or *S.* Typhimurium, respectively. Our results serve to address and deepen our understanding of probiotic action on intestinal porcine epithelial cells and serve as a basis for both human and swine in vivo research and application.

## 2. Materials and Methods

### 2.1. Bacterial Culture

The following bacterial strains were used for our experiments: (1) the probiotic strain *Enterococcus faecium* NCIMB 10415 was acquired from the Hungarian Dairy Experimental Institute Ltd., (2) *E. coli* and (3) *Salmonella* Typhimurium originated from GI infections in pigs. The *E. coli* strain was isolated in 2019 from a clinical sample in Hungary. It expresses F4 fimbriae and produces both heat-stable (STa and STb) and heat-labile (LT) enterotoxins. The *S.* Typhimurium isolate was also obtained from a Hungarian clinical sample (in 2009). All three bacterial strains were preserved on microbank beads at −80 °C.

Cell suspensions were prepared by suspending microbeads in plain DMEM/F12 (without supplementation). Incubation was performed for 18–24 h at 37 °C in the presence of 5% CO_2_/95% air atmosphere in order to mimic the culture conditions of IPEC-J2 cells. In previous experiments, *E. faecium, E. coli* and *S.* Typhimurium were demonstrated to grow to 10^8^ CFU/mL under these circumstances. For cell viability measurements, *E. faecium* suspension of 10^8^, 10^6^, 10^4^ CFU/mL was used. In the pre-, co-, and post-treatment solutions, the applied concentration of *E. faecium* was 10^7^ or 10^8^ CFU/mL and the concentration of *E. coli* and *S.* Typhimurium was 10^8^ CFU/mL. All bacterial suspensions were diluted from the stock solutions (*E. faecium* 10^8^ CFU/mL, *E. coli* 10^6^ CFU/mL, *S Typhimurium* 10^6^ CFU/mL) using plain DMEM/F12 medium (free of antibiotics) as the dilution reagent.

### 2.2. Cell Line and Culture Conditions

The IPEC-J2 epithelial cell line was a kind gift from Dr. Jody Gookin’s Department of Clinical Sciences, College of Veterinary Medicine, North Carolina State University, Raleigh, NC, USA. The cells were grown and maintained in a complete medium consisting of 10 mL of Dulbecco’s Modified Eagle’s Medium and Ham’s F-12 Nutrient Mixture (DMEM/F12) in a 1:1 ratio. This was supplemented with 5% fetal bovine serum (FBS), 5 μg/mL insulin, 5 μg/mL transferrin, 5 ng/mL selenium, 5 ng/mL epidermal growth factor (EGF) and 1% penicillin-streptomycin (Biocenter Ltd., Szeged, Hungary). Cells were cultured at 37 °C in a humidified atmosphere of 5% CO_2_ [26]. Cells with passage numbers 49–52 were used for our experiments. For cell viability determination with the Neutral Red Uptake (NRU) method, cells were cultured onto a 96-well plate (Costar Corning Inc., Corning, NY, USA). For IL-6, IL-8 and intracellular ROS determination, cells were grown on 6-well culture plates (Costar Corning Inc., Corning, NY, USA). For adhesion inhibition, assays cells were seeded onto 24-well cell culture plates (Costar Corning Inc., Corning, NY, USA). For the measurement of paracellular permeability, cells were cultured on 12-well polyester membrane cell culture inserts (Costar Corning Inc., Corning, NY, USA). In each case, cells were cultured until confluency was reached.

In order to remove the remaining antibiotics before starting the treatment of IPEC-J2 cells with the different treatment solutions (described in Section 2.1) IPEC-J2 cells were washed twice with PBS then DMEM/F12 without antibiotics was added to each well, and cells were incubated for 30 min at 37 °C.

### 2.3. Neutral Red Uptake Assay for Cell Viability

The influence of different *E. faecium* bacterial suspension concentrations and different incubation periods on the viability of IPEC-J2 cells were tested with the neutral red uptake method based on the description of Repetto et al. [30]. *E. faecium* suspensions of different concentrations were prepared as described above. IPEC-J2 cells were seeded onto a 96-well plate and incubated with *E. faecium* suspensions of different concentrations (10^8^, 10^6^, 10^4^ CFU/mL) for 1, 2, 4 and 24 h, respectively (37 °C, 5% CO_2_). Treatment with plain medium for 1 h was used as a control in the experiment. The viability of IPEC-J2 cells was measured after 24 h. Absorbance was detected with a Spectramax iD3 instrument (Molecular Devices, San Jose, CA, USA) at a wavelength of 540 nm.

The influence of *E. coli* and *S.* Typhimurium suspensions applied in different concentrations and for different incubation periods was tested by our research group previously [27].

### 2.4. Experimental Setup

**Figure 1:**
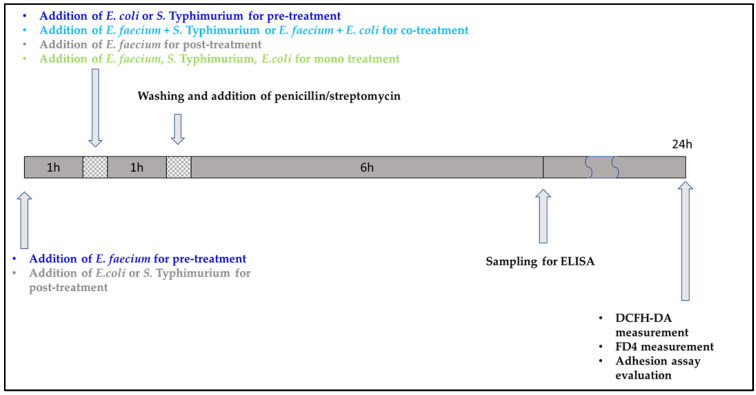
Timeline for experimental setup.

For our DCFH-DA, ELISA, FD4, adhesion assay experiments, IPEC-J2 cells were incubated for 1 h with the pathogen strain *E. coli* or *S.* Typhimurium, respectively. Control cells received plain DMEM/F12 medium. As a positive control, IPEC-J2 cells were mono-incubated with only *E. coli* (10^6^ CFU/mL) or *S.* Typhimurium (10^6^ CFU/mL), respectively. For pre-treatment assays, cells were pre-incubated with *E. faecium* for 1 h before the addition of the pathogen strain. For co-treatment experiments, the pathogen strain (*E. coli* or *S.* Typhimurium) and *E. faecium* were added at the same time to IPEC-J2 cells. In our post-treatment assay, IPEC-J2 cells were incubated with *E. faecium* for 1 h after the treatment with the pathogen strains (*E. coli* or *S*. Typhimurium). Bacterial infections were performed with *E. coli* or *S.* Typhimurium at a concentration of 10^6^ CFU/mL. The applied tolerable pathogen concentration was based on our previous investigations [1]. *E. faecium* suspensions were applied either in a 10^7^ or 10^8^ CFU/ ml concentration, based on our cell viability experimental results. IPEC-J2 cells were also mono-incubated with *E. faecium* 10^8^ and 10^7^ CFU/mL. If further incubation was needed after the treatments, cells were washed with PBS and DMEM/F12 supplemented with antibiotics. Moreover, 1% penicillin-streptomycin was added to prevent the growth of bacteria. The applied treatment solutions in our experiments are summarized in Table 1, and Figure 1 shows the timeline of our experimental setup.

### 2.5. Determination of the Intracellular Redox Status of IPEC-J2 Cells

To evaluate the effect of *E. faecium* on the intracellular redox state of IPEC-J2 cells, the DCFH-DA method was used. IPEC-J2 cells were challenged by *E. coli* or *S.* Typhimurium, respectively. *E. faecium* was added at either 10^8^ CFU/mL or 10^7^ CFU/mL 1 h before (pre-treatment), at the same time (co-treatment) or 1 h after (post-treatment) the indicator *E. coli* (10^8^ CFU/mL) or *S.* Typhimurium (10^8^ CFU/mL) strain was added. Moreover, the effect of *E. faecium* alone (applied in 10^8^ CFU/mL or 10^7^ CFU/mL) on the amount of intracellular reactive oxygen species were tested. As a negative control, cells treated with plain medium were used. Cells treated with either *E. coli* or *S.* Typhimurium served as positive controls. After the treatment, the treatment solutions were discarded and plain medium containing 1% penicillin-streptomycin was added.

Intracellular ROS was measured using 2′, 7′-dichloro-dihydro-fluorescein diacetate (DCFH-DA) dye (Sigma-Aldrich, Budapest, Hungary). DCFH-DA is oxidized to the highly fluorescent form dichloro- fluorescein (DCF) by the intracellular ROS [31]. With this method, the overall oxidative stress is measured in cells, since various free radicals are capable of oxidizing the DCFH-DA.

For the detection, the cells were washed with PBS after 24 h, and DCFH-DA (Sigma-Aldrich, Darmstadt, Germany) reagent (40 mM) was added to the cells. After one hour, the reagent was removed. The cells were then washed twice with phenol-free plain DMEM/F12 (2 mL). Afterward, the cells were scraped and lysed. The lysed cells were then pipetted into an Eppendorf tube and centrifuged for 10 min at 4 °C at 4500 rpm. An amount of 100 μL of supernatant from each sample was added to a 96-well plate. The Spectramax iD3 instrument was used to measure the fluorescence at an excitation wavelength of 480 nm and an emission wavelength of 530 nm.

### 2.6. IL-6 and IL-8 Determination with ELISA

For the ELISA experiments, cells were seeded onto 6-well culture plates and pre-, co-, and post-treatments were performed as described in the experimental setup section. After the removal of the treatments solutions, IPEC-J2 cells were incubated with a cell culture medium and cell supernatants were collected after 6 h. IL-6 and IL-8 secretion were determined by porcine-specific ELISA kits (Sigma-Aldrich, Darmstadt, Germany) according to the manufacturer’s instructions.

### 2.7. Paracellular Permeability Measurements/Assay

The effect of *E. faecium* and *E. coli* or *S.* Typhimurium on the paracellular permeability of IPEC-J2 cells was evaluated with tracer dye FD4 (Sigma-Aldrich, Darmstadt, Germany). Cells were seeded onto 12-well membrane inserts. Prior to treatments, TEER values were measured to check the development of a differentiated confluent monolayer. Mono-, pre-, co-, and post-treatments were performed as described in the section’s experimental setup. After treatment, the cells were washed with PBS and FD4 (dissolved in phenol-free DMEM/F12 medium) at a final concentration of 0.25 mg/mL was added to the apical layer cells. To the basolateral chamber, phenol-free DMEM/F12 medium was added. Cells were incubated at 37 °C (5% CO_2_). Samples of 100 μL were taken from the basolateral chamber after 24 h. The fluorescent signal was measured with a Spectramax iD3 instrument using 485 nm excitation and a 535 nm emission wavelength.

### 2.8. Adhesion Inhibition Assay

In order to evaluate the inhibitory effect of *E. faecium* on *E. coli* or *S.* Typhimurium adhesion to IPEC-J2 cells, *E. faecium* was added at 10^8^ CFU/mL 1 h before (pre-treatment), at the same time (co-treatment) or 1 h after (post-treatment) the indicator *E. coli* or *S.* Typhimurium strain was added. As the control, cells treated with only *E. coli* or *S.* Typhimurium were used. IPEC-J2 cells were incubated for 1 h and then were washed to remove unbound bacteria. The lysis of cells was performed with 500 µL 0.1% Triton X-100 (Sigma-Aldrich, Darmstadt, Germany). Viable *E. coli* and *S.* Typhimurium counts were determined by serial dilution and plating on ChromoBio Coliform (for *E. coli*) or ChromoBio Salmonella Plus Base (for *S.* Typhimurium) agar. ChromoBio Coliform and ChromoBio Salmonella Plus Base selective agars were purchased from Biolab Zrt. (Budapest, Hungary). Adhesion was calculated as the control percentage. Adhering *E. coli* and *S.* Typhimurium was normalized to the control.

### 2.9. Statistical Analysis

Data were tested for normality of distribution and statistical analysis was performed with the R 4.0.4 software package. The data are given as mean values ± S.E.M (n) where n refers to the number of parallel measurements. Differences between means were evaluated by one-way analysis of variance (ANOVA) with a post hoc Tukey’s test when data were of normal distribution and homogeneity of variances was confirmed, or a Kruskal–Wallis nonparametric test. A *p* value of <0.05 was accepted to indicate statistical significance. The exact statistical comparisons are indicated in the text and in the appropriate figure legends.

## 3. Results

### 3.1. Cell Viability Assay

**Figure 2:**
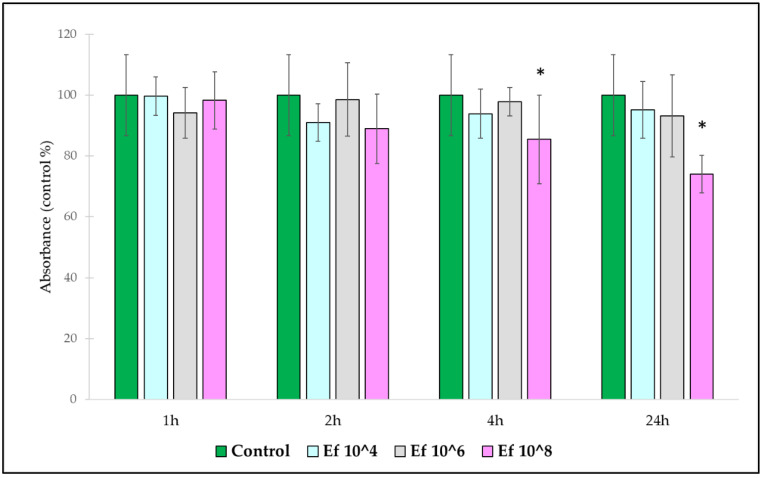
Viability of IPEC-J2 cells after treatment with *E. faecium* NCIMB 10415 for different times. **Control**: plain cell culture medium treatment for 1 h; **1 h, Ef 10^4**: treatment for 1 h with *E. faecium* suspension of 10^4^ CFU/mL; **1 h, Ef 10^6**: treatment for 1 h with *E. faecium* suspension of 10^6^ CFU/mL; **1 h, Ef 10^8**:treatment for 1 h with *E. faecium* suspension of 10^8^ CFU/mL; **2 h, Ef 10^4**:treatment for 2 h with *E. faecium* suspension of 10^4^ CFU/mL; **2 h, Ef 10^6**: treatment for 2 h with *E. faecium* suspension of 10^6^ CFU/mL; **2 h, Ef 10^8**: treatment for 2 h with *E. faecium* suspension of 10^8^ CFU/mL; **4 h, Ef 10^4**: treatment for 4 h with *E. faecium* suspension of 10^4^ CFU/mL; **4 h, Ef 10^6**: treatment for 4 h with *E. faecium* suspension of 10^6^ CFU/mL; **4 h, Ef 10^8**: treatment for 4 h with *E. faecium* suspension of 10^8^ CFU/mL; **24 h, Ef 10^4**: treatment for 24 h with *E. faecium* suspension of 10^4^ CFU/mL; **24 h, Ef 10^6**: treatment for 24 h with *E. faecium* suspension of 10^6^ CFU/mL; **24 h, Ef 10^8**: treatment for 24 h with *E. faecium* suspension of 10^8^ CFU/mL. Data are shown as means with standard deviations, n = 6/group. * Indicates significant differences (*p* ≤ 0.05) compared to the control.

In order to determine the effect of *E. faecium* suspensions on the viability of IPEC-J2 cells, the neutral red uptake method was used. *E. faecium* suspensions of a 10^8^ CFU/mL concentration significantly reduced the viability of IPEC-J2 cells when they were applied for 4 and 24 h (Figure 2). Any other treatment concentrations and treatment times did not cause any significant change in the viability of IPEC-J2 cells as compared to the control. The cytotoxic effect of *E. coli* and *S.* Typhimurium were previously tested, the optimal treatment concentrations were found to be 10^6^ CFU/mL and the optimal treatment time was set to 1 h [1].

### 3.2. Effect of Enterococcus faecium on the Intracellular Redox State of IPEC-J2 Cells Challenged by Salmonella Typhimurium and Escherichia coli

**Figure 3:**
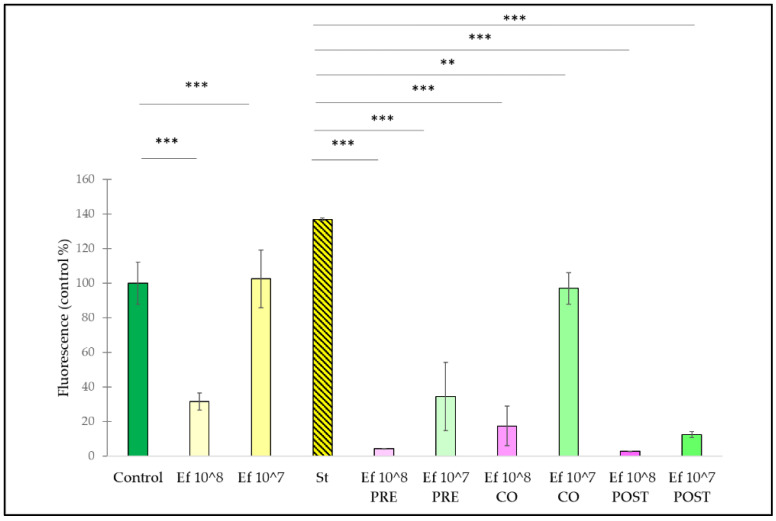
Amount of intracellular ROS after treatment with *S.* Typhimurium and *E. faecium* and their combinations. *E. faecium* was added 1 h before (pre-treatment), at the same time (co-treatment) or after (post-treatment) the addition of *S.* Typhimurium. *E. faecium* was added in 10^8^ CFU/mL or in 10^7^ CFU/mL concentration. **Control**: plain cell culture medium treatment; **St**: *S.* Typhimurium 10^6^ CFU/mL; **Ef 10^8**: *E. faecium* 10^8^ CFU/mL; **Ef 10^7**: *E. faecium* 10^7^ CFU/mL; **Ef 10^8 PRE**: pre-treatment with *E. faecium* 10^8^ CFU/mL + *S.* Typhimurium 10^6^ CFU/mL; **Ef 10^7 PRE**: pre-treatment with *E. faecium* 10^7^ CFU/mL + *S.* Typhimurium 10^6^ CFU/mL; **Ef 10^8 CO**: co-treatment with *E. faecium* 10^8^ CFU/mL + *S.* Typhimurium 10^6^ CFU/mL; **Ef 10^7 CO**: co-treatment with *E. faecium* 10^7^ CFU/mL + *S.* Typhimurium 10^6^ CFU/mL; **Ef 10^8 POST**: post-treatment with *E. faecium* 10^8^ CFU/mL + *S.* Typhimurium 10^6^ CFU/mL; **Ef 10^7 POST**: post-treatment with *E. faecium* 10^7^ CFU/mL + *S.* Typhimurium 10^6^ CFU/mL. Data are shown as means with standard deviations, n = 6/group. ** *p* ≤ 0.01; *** *p* ≤ 0.0001.

In order to characterize the intracellular redox state of the IPEC-J2 cells, the DCFH-DA method was used. Treatment with *S.* Typhimurium caused an increase in the fluorescence compared to the control (Figure 3). All three treatment combinations (i.e., pre-treatment, co-treatment and post-treatment with *S.* Typhimurium and *E. faecium* in two different concentrations) resulted in a decreased amount of ROS. When IPEC-J2 cells were treated with only *E. faecium* 10^8^ CFU/mL and 10^7^ CFU/mL, a decrease in fluorescence could be observed compared to the control.

**Figure 4:**
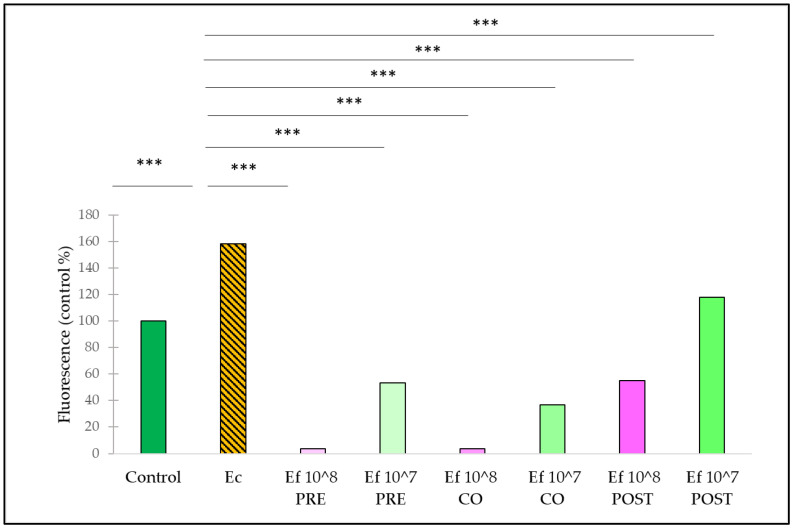
Amount of intracellular ROS after treatment with *E. coli* and *E. faecium*. *E. faecium* was added 1 h before (pre-treatment), at the same time (co-treatment) or after (post-treatment) the addition of *E. coli*. *E. faecium* was added in 10^8^ CFU/mL or in 10^7^ CFU/mL concentration. **Control**: plain cell culture medium treatment; **Ec**: *E. coli* 10^6^ CFU/mL; **Ef 10^8 PRE**: pre-treatment with *E faecium* 10^8^ CFU/mL + *E. coli* 10^6^ CFU/mL; **Ef 10^7 PRE**: pre-treatment with *E. faecium* 10^7^ CFU/mL + *E. coli* 10^6^ CFU/mL; **Ef 10^8 CO**: co-treatment with *E. faecium* 10^8^ CFU/mL + *E. coli* 10^6^ CFU/mL; **Ef 10^7 CO**: co-treatment with *E. faecium* 10^7^ CFU/mL + *E. coli* 10^6^ CFU/mL; **Ef 10^8 POST**: post-treatment with *E. faecium* 10^6^ CFU/mL + *E. coli* 10^6^ CFU/mL; **Ef 10^7 POST**: post-treatment with *E. faecium* 10^7^ CFU/mL + *E. coli* 10^6^ CFU/mL. Data are shown as means with standard deviations, n = 6/group. *** *p* ≤ 0.0001.

Treatment with *E. coli* caused an increase in the fluorescence compared to the control (Figure 4). The pre-treatment with *E. faecium* significantly reduced the amount of reactive oxygen species in the cells compared with samples only treated with *E. coli*. Both applied concentrations (10^8^ CFU/mL and 10^7^ CFU/mL) of *E. faecium* resulted in a significant decrease in reactive oxygen species. The same could be observed in the case of co-treatments and post-treatments.

### 3.3. Effect of E. faecium on IL-6 and IL-8 Production of IPEC-J2 Cells Provoked by E. coli or S. Typhimurium

**Figure 5:**
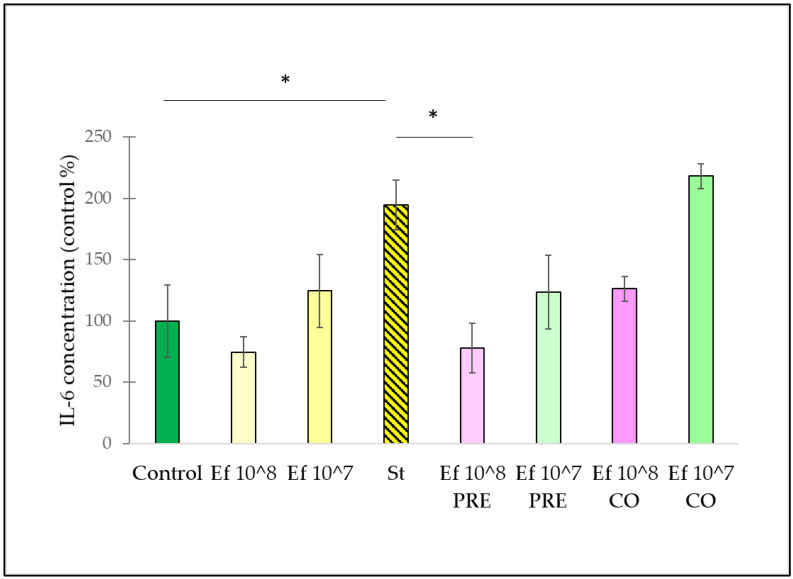
Induction of IL-6 secretion of IPEC-J2 cells after stimulation with *S.* Typhimurium and *E. faecium*. *E. faecium* was added 1 h before (pre-treatment) or at the same time (co-treatment) of the addition of S. Typhimurium. *E. faecium* was added in 10^8^ CFU/mL or in 10^7^ CFU/mL concentration. **Control**: plain cell culture medium treatment; **St**: *S.* Typhimurium 10^6^ CFU/mL; **Ef 10^8**: *E. faecium* 10^8^ CFU/mL; **Ef 10^7**: *E. faecium* 10^7^ CFU/mL**; Ef 10^8 PRE**: pre-treatment with *E. faecium* 10^8^ CFU/mL + *S.* Typhimurium 10^6^ CFU/mL; **Ef 10^7 PRE**: pre-treatment with *E. faecium* 10^7^ CFU/mL + *S.* Typhimurium 10^6^ CFU/mL; **Ef 10^8 CO**: co-treatment with *E. faecium* 10^8^ CFU/mL + *S.* Typhimurium 10^6^ CFU/mL; **Ef 10^7 CO**: co-treatment with *E. faecium* 10^7^ CFU/mL + *S.* Typhimurium 10^6^ CFU/mL. Data are shown as means with standard deviations, n = 6/group. * *p* ≤ 0.05.

Infection of intestinal epithelial cells with *S.* Typhimurium significantly induced the secretion of IL-6 compared to the controls (i.e., non-infected cells) (Figure 5). In comparison, treatment with only the probiotic strain did not result in a significant change in IL-6 secretion, even if *E. faecium* was applied at a concentration of 10^8^ CFU/mL or 10^7^ CFU/mL. The pre-treatment with *E. faecium* 10^8^ CFU/mL caused a significant decrease in IL-6 production as compared to the IL-6 secretion induced by *S.* Typhimurium. However, the co-treatment of *S.* Typhimurium and *E. faecium* at 10^8^ CFU/mL did not alter the IL-6 secretion compared to the IL-6 secretion evoked by *S.* Typhimurium. The pre-treatment and the co-treatment with *E. faecium* 10^7^ CFU/mL failed to significantly decrease IL-6 secretion compared to the IL-6 production induced by *S.* Typhimurium.

**Figure 6:**
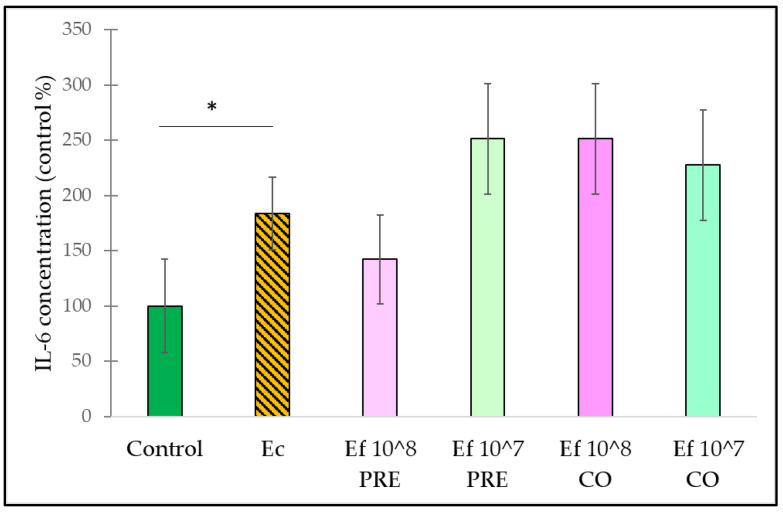
Induction of IL-6 secretion of IPEC-J2 cells after stimulation with *E. coli* and *E. faecium*. *E. faecium* was added 1 h before (pre-treatment) or at the same time (co-treatment) of the addition of *E. coli*. *E. faecium* was added in 10^8^ CFU/mL or in 10^7^ CFU/mL concentration. **Control**: plain cell culture medium treatment; **Ec**: *E. coli* 10^6^ CFU/mL; **Ef 10^8 PRE**: pre-treatment with *E. faecium* 10^8^ CFU/mL + *E. coli* 10^6^ CFU/mL; **Ef 10^7 PRE**: pre-treatment with *E. faecium* 10^7^ CFU/mL + *E. coli* 10^6^ CFU/mL; **Ef 10^8 CO**: co-treatment with *E. faecium* 10^8^ CFU/mL + *E. coli* 10^6^ CFU/mL; **Ef 10^7 CO**: co-treatment with *E. faecium* 10^7^ CFU/mL + *E. coli* 10^6^ CFU/mL. Data are shown as means with standard deviations, n = 6/group. * *p* ≤ 0.05.

IL-6 secretion was induced significantly by *E. coli* in comparison to the control cells. Neither pre-treatment nor co-treatment with *E. faecium* could compensate for the IL-6 elevation induced by *E. coli* (Figure 6).

**Figure 7:**
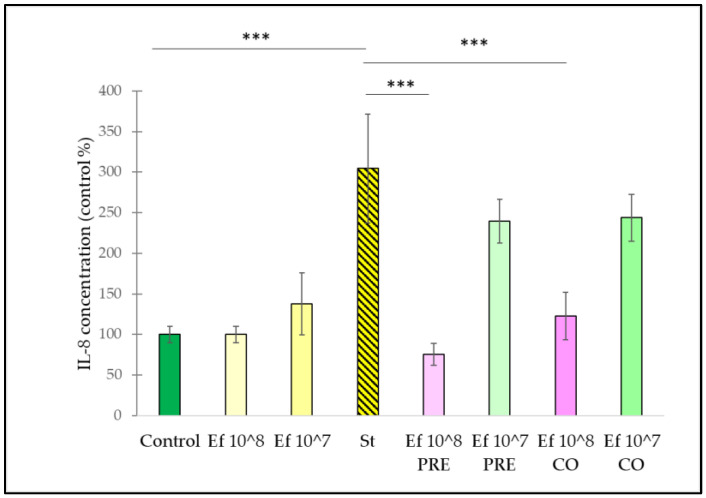
Induction of IL-8 secretion of IPEC-J2 cells after stimulation with *S.* Typhimurium and *E. faecium*. *E. faecium* was added 1 h before (pre-treatment) or at the same time (co-treatment) of the addition of *S.* Typhimurium. *E. faecium* was added in 10^8^ CFU/mL or in 10^7 CFU/mL concentration. **Control**: plain cell culture medium treatment; **St**: *S.* Typhimurium 10^6^ CFU/mL; **Ef 10^8**: *E. faecium* 10^8^ CFU/mL; **Ef 10^7**: *E. faecium* 10^7^ CFU/mL; **Ef 10^8 PRE**: pre-treatment with *E. faecium* 10^8^ CFU/mL + *S.* Typhimurium 10^6^ CFU/mL; **Ef 10^7 PRE**: pre-treatment with *E. faecium* 10^7^ CFU/mL + *S.* Typhimurium 10^6^ CFU/mL; **Ef 10^8 CO**: co-treatment with *E. faecium* 10^8^ CFU/mL + *S.* Typhimurium 10^6^ CFU/mL; **Ef 10^7 CO**: co-treatment with *E. faecium* 10^7^ CFU/mL + *S.* Typhimurium 10^6^ CFU/mL. Data are shown as means with standard deviations, n = 6/group; *** *p* ≤ 0.0001.

Infection of IPEC-J2 cells with *S.* Typhimurium also increased the secretion of IL-8 (Figure 7). Treatment with the probiotic strain itself did not result in a significant change in IL-8 secretion, regardless of the applied concentration. Pre-treatment and co-treatment with *E. faecium*, applied at a concentration of 10^8^ CFU/mL, significantly reduced the secretion of IL-8 compared to the amount of IL-8 secretion when IPEC-J2 cells were challenged by *S.* Typhimurium. Pre-treatment and co-treatment with *E. faecium*, applied at a concentration of 10^7^ CFU/mL, failed to decrease the IL-8 secretion in comparison to the secretion observed when cells were treated with *S.* Typhimurium itself.

**Figure 8:**
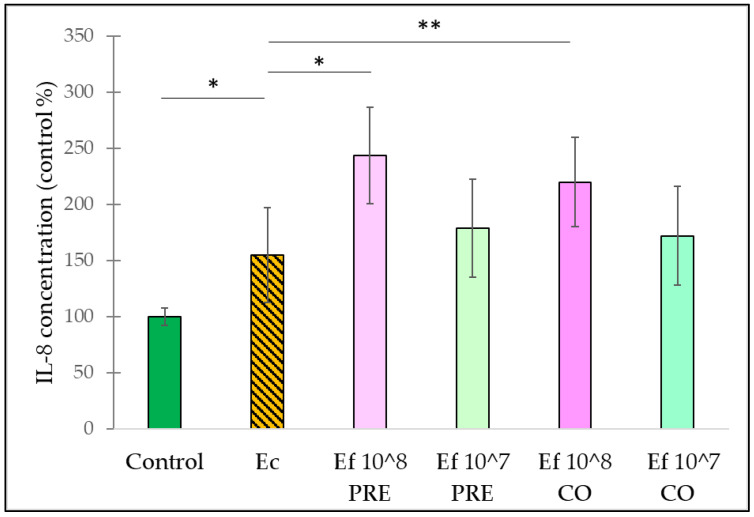
Induction of IL-8 secretion of IPEC-J2 cells after stimulation with *E. coli* and *E. faecium*. *E. faecium* was added 1 h before (pre-treatment) or at the same time (co-treatment) of the addition of *E. coli*. *E. faecium* was added in 10^8^ CFU/mL or in 10^7^ CFU/mL concentration. **Control**: plain cell culture medium treatment; **Ec**: *E. coli* 10^6^ CFU/mL; **Ef 10^8 PRE**: pre-treatment with *E. faecium* 10^8^ CFU/mL + *E. coli* 10^6^ CFU/mL; **Ef 10^7 PRE**: pre-treatment with *E. faecium* 10^7^ CFU/mL + *E. coli* 10^6^ CFU/mL; **Ef 10^8 CO**: co-treatment with *E. faecium* 10^8^ CFU/mL + *E. coli* 10^6^ CFU/mL; **Ef 10^7 CO**: co-treatment with *E. faecium* 10^7^ CFU/mL + *E. coli* 10^6^ CFU/mL. Data are shown as means with standard deviations, n = 6/group. * *p* ≤ 0.05; ** *p* ≤ 0.01.

IL-8 secretion was induced significantly by *E. coli* compared to the control cells (Figure 8). Pre-treatment and co-treatment with *E. faecium*, applied at a concentration of 10^8^ CFU/mL further increased the secretion of IL-8. The pre-treatment and co-treatment with *E. faecium*, applied at a concentration of 10^7^ CFU/mL, failed to cause any significant effect on IL-8 secretion.

### 3.4. Effect of E. faecium on the Adhesion of S. Typhimurium and E. coli to IPEC-J2 Cells

**Figure 9:**
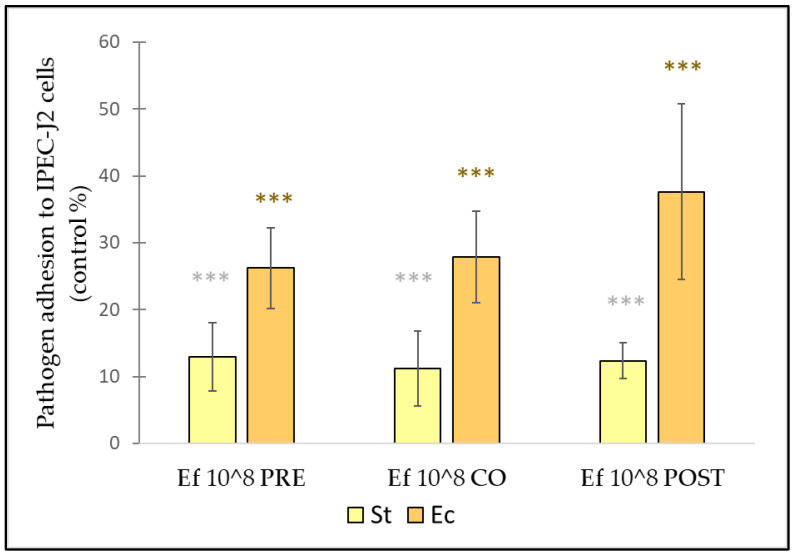
Inhibitory effect of *E. faecium* on *E. coli* and *S.* Typhimurium adhesion to IPEC-J2 cells. *E. coli* and *S.* Typhimurium adhesion inhibitions were determined upon incubation with *E. faecium* added 1 h before (pre-treatment), at the same time (co-treatment) and 1 h after (post-treatment) the addition of *E. coli* and *S.* Typhimurium, respectively. *E. faecium* was added in 10^8^ CFU/mL. **Ec**: *E. coli* 10^6^ CFU/mL**; Ef PRE**: pre-treatment with *E. faecium* 10^8^ CFU/mL + *E. coli* or *S.* Typhimurium 10^6^ CFU/mL; **Ef CO**: co-treatment with *E. faecium* 10^8^ CFU/mL + *E. coli* or *S.* Typhimurium 10^6^ CFU/mL; **Ef POST**: post-treatment with *E. faecium* 10^8^ CFU/mL + *E. coli* or *S.* Typhimurium 10^6^ CFU/mL. Values are presented as means ± SEs of four independent experiments. *** *p* ≤ 0.0001 compared to treatment with *S.* Typhimurium. *** *p* ≤ 0.0001 compared to treatment with *E. coli*.

*E. faecium* was able to inhibit the adhesion of both *E. coli* and *S.* Typhimurium in all treatment combinations (Figure 9). When IPEC-J2 cells were challenged by *E. coli*, pre-treatment with *E. faecium* had the highest inhibitory effect, followed by co-treatment, while post-treatment showed the lowest inhibitory effect. *E. coli* adhesion was 26.2% in the case of pre-treatment, 27.8% in the co-treatment assay and 37.6% in the post-treatment. When IPEC-J2 cells were exposed to *S.* Typhimurium, only a minor difference could be found in the effect of adhesion between the different treatment (pre-, co- and post-) conditions. *S.* Typhimurium adhesion was 12.9% in the case of pre-treatment, 11.2% in the co-treatment assay, and 12.3% for the post-treatment.

### 3.5. The Effect of E. faecium on Paracellular Permeability of IPEC-J2 Cells Challenged by E. coli and S. Typhimurium

**Figure 10:**
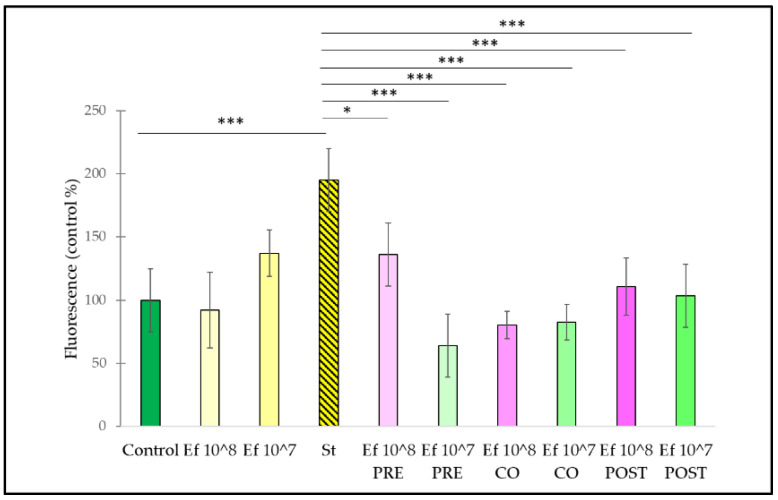
Effect of *E. faecium* on the paracellular permeability of IPEC-J2 cells treated with *S.* Typhimurium. *E. faecium* was added 1 h before (pre-treatment), at the same time (co-treatment) and 1 h after (post-treatment) the addition of *S.* Typhimurium. Detection of the FD4 dye was performed 24 after the treatment of *S.* Typhimurium. **Control**: plain cell culture medium treatment; **Ef 10^8**: *E. faecium* 10^8^ CFU/mL; **Ef 10^7**: *E. faecium* 10^7^ CFU/mL**; Ef 10^8 PRE**: pre-treatment with *E. faecium* 10^8^ CFU/mL + *S.* Typhimurium 10^6^ CFU/mL; **Ef 10^7 PRE**: pre-treatment with *E. faecium* 10^7^ CFU/mL + *S.* Typhimurium 10^6^ CFU/mL; **Ef 10^8 CO**: co-treatment with *E. faecium* 10^8^ CFU/mL + *S.* Typhimurium 10^6^ CFU/mL; **Ef 10^7 CO**: co-treatment with *E. faecium* 10^7^ CFU/mL + *S.* Typhimurium 10^6^ CFU/mL. **Ef 10^8 POST**: post-treatment with *E. faecium* 10^8^ CFU/mL + *S.* Typhimurium 10^6^ CFU/mL; **Ef 10^7 POST**: post-treatment with *E. faecium* 10^7^ CFU/mL + *S.* Typhimurium 10^6^ CFU/mL. Data are shown as means ± SEs of three independent experiments. * *p* ≤ 0.05; *** *p* ≤ 0.0001 compared to treatment with *S.* Typhimurium.

**Figure 11:**
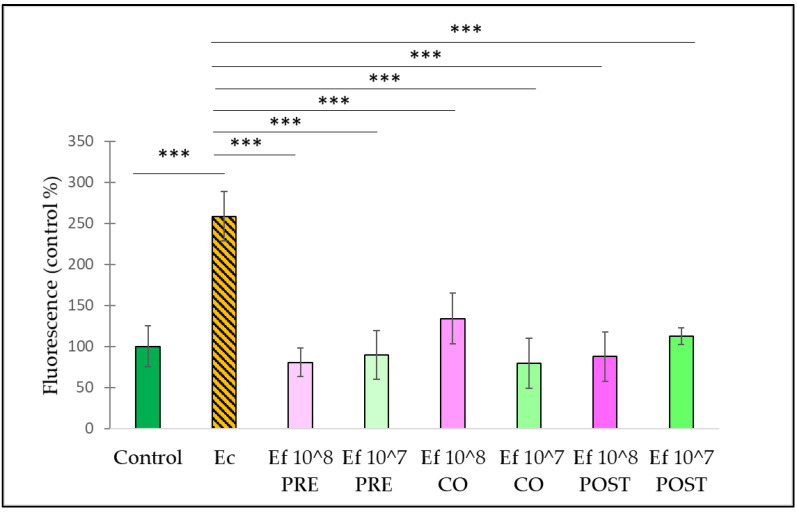
Effect of *E. faecium* on the paracellular permeability of IPEC-J2 cells treated with *E. coli*. *E. faecium* was added 1 h before (pre-treatment), at the same time (co-treatment) and 1 h after (post-treatment) the addition of *E. coli*. Detection of the FD4 dye was performed 24 after the treatment of *E. coli*. **Control**: plain cell culture medium treatment; **Ec**: *E. coli* 10^6^ CFU/mL**; Ef 10^8 PRE**: pre-treatment with *E. faecium* 10^8^ CFU/mL + *E. coli* 10^6^ CFU/mL; **Ef 10^7 PRE**: pre-treatment with *E. faecium* 10^7^ CFU/mL + *E. coli* 10^6^ CFU/mL; **Ef 10^8 CO**: co-treatment with *E. faecium* 10^8^ CFU/mL + *E. coli* 10^6^ CFU/mL; **Ef 10^7 CO**: co-treatment with *E. faecium* 10^7^ CFU/mL + *E. coli* 10^6^ CFU/mL; **Ef 10^8 POST**: post-treatment with *E. faecium* 10^8^ CFU/mL + *E. coli* 10^6^ CFU/mL; **Ef 10^7 POST**: post-treatment with *E. faecium* 10^7^ CFU/mL + *E. coli* 10^6^ CFU/mL. Data are shown as means ± SEs of three independent experiments; *** *p* ≤ 0.0001 compared to treatment with *E. coli*.

After 24 h of pathogen exposure, the epithelial cell layer was partially disrupted. The fluorescence intensity measured in the basolateral compartment significantly increased (compared to the untreated control samples) when IPEC-J2 cells were treated with *S.* Typhimurium (Figure 10) or *E. coli* (Figure 11). The treatment with *E. faecium* alone, in two different concentrations (10^8^ CFU/mL or 10^7^ CFU/mL), did not result in the alteration of fluorescence intensity (Figure 10). Pre-treatment, co-treatment and post-treatment with *E. faecium* significantly decreased the presence of FD4 tracer in the basolateral chamber, when cells were exposed to *S.* Typhimurium (Figure 10). The same effect could be observed when IPEC-J2 cells were challenged by *E. coli* (Figure 11).

## 4. Discussion

The present study aims to elucidate the effect of *E. faecium* on the inflammatory response, internal redox state and barrier function of the intestinal epithelium. In addition, the adhesion inhibiting effects of *E. faecium* on *S.* Typhimurium and *E. coli* were investigated. In order to examine the capability of the probiotic strain to modify the epithelial response to a pathogenic challenge, epithelial cells were incubated with *E. faecium* and either *E. coli* or *S.* Typhimurium. Our hypothesis was that *E. faecium* might (1) reduce the secretion of proinflammatory cytokines, (2) decrease the amount of reactive oxygen species, (3) improve epithelial integrity and (4) inhibit the adhesion of pathogenic bacteria.

### 4.1. Inflammatory Response

Intestinal epithelial cells play a major role in activating the adaptive immune response upon pathogen infection, mostly by producing various cytokines [32,33,34,35,36,37,38]. In our experiments, both IL-6 and IL-8 secretion were significantly increased when IPEC-J2 cells were challenged by *E. coli* or *S.* Typhimurium, respectively. These findings agree with previous studies that also demonstrated an increase in IL-6 or IL-8 upon pathogen challenge [20,32]. The pre-treatment with *E. faecium* in a concentration of 10^8^ CFU/mL could abrogate the increase in both IL-6 and IL-8 secretion, while the co-incubation with *E. faecium* applied at a concentration of 10^8^ CFU/mL could also significantly decrease the secretion of IL-8 when an inflammatory response was evoked by *S.* Typhimurium. *Salmonella*-induced IL-8 secretion was decreased by probiotic strains *Lactobacillus reuteri* ATCC 53608 and *Bacillus licheniformis* ATCC 10716, which agree with our finding, that probiotics may attenuate the proinflammatory cytokine response upon pathophysiological challenge [17]. When IPEC-J2 cells were challenged with *E. coli*, the pre- and co-incubation with 10^8^ CFU/mL *E. faecium* either did not show any effect on the production of proinflammatory cytokines (IL-6) or unexpectedly, further increased their secretion (IL-8). Others, however, found that the *E. coli* induced IL-8 elevation was reduced by *E. faecium* co-incubation [20,24]. This inconsistency might be due to differences in the mode of action of various probiotic strains [17].

### 4.2. Response to Oxidative Stress

Here, *E. coli* and *S.* Typhimurium were used to induce oxidative stress in IPEC-J2 cells. The exact mechanism of how *E. coli* and *Salmonella* exert their oxidative stress-inducing effect is obscure, but pathogens may produce oxygen to generate an aerobic environment, thus establishing oxidative stress conditions in the intestines [8]. To confirm the antioxidant effect of the application of *E. faecium* as a pre-treatment, co-treatment, and post-treatment, we determined the capacity of the treatment methods for the alleviation of ROS production. *E. coli* and *S.* Typhimurium induced an intracellular ROS burst in IPEC-J2 cells. Pre-, co-, and post-treatment with *E. faecium* applied in either 10^8^ CFU/mL or 10^7^ CFU/mL remarkably reduced ROS generation induced by *E. coli* or *S.* Typhimurium, respectively. This finding indicates that *E. faecium* could alleviate the oxidative stress caused by *E. coli* and *S.* Typhimurium. Interestingly, certain probiotics have been shown to mitigate induced ROS production, and that pre-treatment of IPEC-J2 cells with *L. plantarum* ZLP001 reduced the ROS burst evoked by H_2_O_2_ in IPEC-J2 cells [8], supporting the potential beneficial effect of probiotics on ROS generation.

### 4.3. Pathogen Adhesion

The inhibition of pathogen adhesion is one of the most important properties in how probiotics may exert their beneficial effects. The ability of different probiotic strains to inhibit pathogen adhesion has been studied extensively [39,40]. Our results confirm that probiotics can inhibit pathogen adhesion. However, in our experiments, the inhibition effect of *E. faecium* was independent of the time of addition. Significant adhesion inhibition was observed in the case of all three treatment conditions, similar to other recent reports [41]. Our finding that post-treatment could also inhibit the adhesion of both *E. coli* and *S.* Typhimurium indicates that *E. faecium* was able to disrupt established pathogen colonization.

### 4.4. Epithelial Barrier Function

One mode of action of probiotics is likely the strengthening of the epithelial barrier [7]. *E. coli* and *S.* Typhimurium can disrupt this barrier integrity. The enhancement of intestinal barrier function by probiotics has been intensely investigated [7]. In our experiments, the FD4 method was used to assess the changes in the integrity and permeability of the epithelial barrier. In our experiments, *E. faecium* alone had no significant effect on the amount of FD4 dye measured in the basolateral compartment. This result agrees with studies showing that the use of probiotics alone does not affect the integrity and permeability of the epithelial barrier [21,42,43]. However, other in vitro studies showed that the application of probiotic bacteria alone might enhance the barrier function [44,45,46]. Interestingly, *E. coli* or *S.* Typhimurium’s induced pathophysiological challenge resulted in a significant increase in the amount of FD4 dye measured in the basolateral compartment, indicating that these strains were able to disrupt the integrity of the barrier, in line with previous findings [47]. Lipopolysaccharides or bacterial metabolites might be responsible for the disruption of the epithelial barrier [21]. Pathogens might also induce the apoptosis of enterocytes, which results in increased TEER values, indicating that the barrier function has been damaged. We suggest that *E. faecium* might be able to counteract the increased FD4 flux. Studies on Caco-2 and T84 cells have shown that probiotic bacteria could prevent the barrier disrupting effects of *E. coli* [42,48]. Our experiments showed that pre-treatment, co-treatment, and post-treatment with *E. faecium* could also prevent the damaging effects on barrier integrity induced by *E. coli* or *S.* Typhimurium, and significantly reduce the FD4 flux.

Taken together, the treatment of IPEC-J2 cells with *E. faecium* has multiple beneficial effects on cell integrity, paracellular permeability and intracellular ROS production, proinflammatory cytokine secretions, and the adhesion of *Salmonella* Typhimurium and *Escherichia coli*. Therefore, we suggest that *E. faecium* is a promising probiotic candidate for both human and animal use. The use of this strain as a probiotic also addresses the challenge of finding alternative treatments that can strengthen gastrointestinal health without the use of antibiotics. Furthermore, our in vitro model proved to be a useful tool to examine the effects of promising probiotics and other alternative substance candidates in future investigations.

## Figures and Tables

**Table 1:** Applied treatment types in the co-culture experiments.

Type of Treatment	Applied Probiotic Strain and Concentration	Applied Pathogen Strain and Concentration
pre-addition *E. faecium* + *S.* Typhimurium	*E. faecium* 10^7^ or 10^8^ CFU/mL prior to infection	*S.* Typhimurium 10^6^ CFU/mL
co-addition *E. faecium* + *S.* Typhimurium	*E. faecium* 10^7^ or 10^8^ CFU/mL at the same time with infection	*S.* Typhimurium 10^6^ CFU/mL
post-addition *E. faecium* + *S.* Typhimurium	*E. faecium* 10^7^ or 10^8^ CFU/mL after infection	*S.* Typhimurium 10^6^ CFU/mL
pre- addition *E. faecium* + *E. coli*	*E. faecium* 10^7^ or 10^8^ CFU/mL prior to infection	*E. coli* 10^6^ CFU/mL
Co-addition *E. faecium* + *E. coli*	*E. faecium* 10^7^ or 10^8^ CFU/mL at the same time with infection	*E. coli* 10^6^ CFU/mL
Post-addition *E. faecium* + *E. coli*	*E. faecium* 10^7^ or 10^8^ CFU/mL after infection	*E. coli* 10^6^ CFU/mL
*E. faecium* 10^7^ (mono-incubation)	*E. faecium* 10^7^ CFU/mL	-
*E. faecium* 10^8^ (mono-incubation)	*E. faecium* 10^8^ CFU/mL	-
*S.* Typhimurium (mono-incubation)	-	*S.* Typhimurium 10^6^ CFU/mL
*E. coli* (mono-incubation)	-	*E. coli* 10^6^ CFU/mL

## Data Availability

All data that supports the above-detailed findings can be obtained from the corresponding author upon request.
